# Gastrointestinal Helminth Infection Improves Insulin Sensitivity, Decreases Systemic Inflammation, and Alters the Composition of Gut Microbiota in Distinct Mouse Models of Type 2 Diabetes

**DOI:** 10.3389/fendo.2020.606530

**Published:** 2021-02-05

**Authors:** Zainab Khudhair, Rafid Alhallaf, Ramon M. Eichenberger, Jen Whan, Andreas Kupz, Matt Field, Lutz Krause, David T. Wilson, Norelle L. Daly, Paul Giacomin, Javier Sotillo, Alex Loukas

**Affiliations:** ^1^Centre for Molecular Therapeutics, Australian Institute of Tropical Health and Medicine, James Cook University, Cairns, QLD, Australia; ^2^Advanced Analytical Center, James Cook University, Cairns, QLD, Australia; ^3^John Curtin School of Medical Research, Australian National University, Canberra, ACT, Australia; ^4^Microba Pty Ltd, Brisbane, QLD, Australia; ^5^Parasitology Reference and Research Laboratory, Centro Nacional de Microbiología, Instituto de Salud Carlos III, Madrid, Spain

**Keywords:** type 2 diabetes, *Nippostrongylus brasiliensis*, helminth, eosinophils, M2 macrophages, high glycemic index diet, high fat diet, microbiota

## Abstract

Type 2 diabetes (T2D) is a major health problem and is considered one of the top 10 diseases leading to death globally. T2D has been widely associated with systemic and local inflammatory responses and with alterations in the gut microbiota. Microorganisms, including parasitic worms and gut microbes have exquisitely co-evolved with their hosts to establish an immunological interaction that is essential for the formation and maintenance of a balanced immune system, including suppression of excessive inflammation. Herein we show that both prophylactic and therapeutic infection of mice with the parasitic hookworm-like nematode, *Nippostrongylus brasiliensis*, significantly reduced fasting blood glucose, oral glucose tolerance and body weight gain in two different diet-induced mouse models of T2D. Helminth infection was associated with elevated type 2 immune responses including increased eosinophil numbers in the mesenteric lymph nodes, liver and adipose tissues, as well as increased expression of *IL-4* and alternatively activated macrophage marker genes in adipose tissue, liver and gut. *N. brasiliensis* infection was also associated with significant compositional changes in the gut microbiota at both the phylum and order levels. Our findings show that *N. brasiliensis* infection drives changes in local and systemic immune cell populations, and that these changes are associated with a reduction in systemic and local inflammation and compositional changes in the gut microbiota which cumulatively might be responsible for the improved insulin sensitivity observed in infected mice. Our findings indicate that carefully controlled therapeutic hookworm infection in humans could be a novel approach for treating metabolic syndrome and thereby preventing T2D.

## Introduction

Diabetes is a metabolic disease resulting from the absence of, or deficiency in, insulin secretion, insulin action or both, leading to an abnormal metabolism of carbohydrates and elevated levels of glucose in the blood (1). The main types of diabetes are type 1 (T1D), which represents around 10% of all diabetes cases, and type 2 (T2D), which represents around 90% of all diabetes globally. Diabetes is a fast-growing health problem worldwide. According to the International Diabetes Federation there were 424.9 million people living with diabetes, and a further 352.1 million with impaired glucose tolerance in 2017 (1). Diabetes caused 4 million deaths and accounted for 10.7% of global all-cause mortality and cost USD 727 billion in healthcare spending in 2017 alone (1).

Cumulative evidence suggests that T2D is associated with inflammation. Induction of T helper 1 (Th1) immune responses, in particular activation of M1 macrophages (MACs) and increased production of pro-inflammatory cytokines such as IL-1β, IFN-γ, TNF-α, and IL-6 play a crucial role in the destruction of pancreatic β-cells, and insulin resistance in adipose tissue (AT), liver and muscle (2). In contrast, cells such as type 2 innate lymphoid cells (ILC2s), eosinophils, and M2 MACs, as well as increased levels of Th2 cytokines such as IL-5, IL-4, and IL-13 have been found to regulate adipose tissue homeostasis (3, 4), liver regeneration (5), and gastrointestinal homeostasis (6), leading to whole body metabolic homeostasis. Moreover, regulation of metabolic homeostasis and inflammation in obesity, metabolic syndrome and T2D has been increasingly connected with the gut microbiota (7). Disturbance of the intestinal microbial community leads to altered immune responses that can result in various inflammatory disorders (8).

Environmental changes such as altered dietary habits, improved sanitation, vaccination and excessive use of antibiotics has reduced our exposure to various infectious agents and symbiotic microorganisms that had a co-evolutionary relationship with humans (9). This relationship has established an immunological interaction with highly developed regulatory pathways that serve to dampen inappropriate immune responses, which are considered the key drivers in many immune-mediated disorders, including T1D (10). Helminth infections induce Th2 immune responses by expansion of innate immune cells such as eosinophils, M2 MACs, ILCs, and upregulation of cytokines such as IL-4, IL-5, and IL-13. Furthermore, it has been widely shown that helminth infections promote expansion and/or recruitment of regulatory T cells (Tregs) that play an important role in regulating inflammation (11). Recent experimental evidence in animal models has highlighted the therapeutic role of helminth-mediated induction of Th2- and Treg-mediated immune responses in many inflammatory diseases such as inflammatory bowel disease (IBD), multiple sclerosis (MS), rheumatoid arthritis, asthma and T1D (12). Likewise, helminth infections have shown promising results as a therapeutic strategy in human subjects with IBD, celiac disease and MS (13–16).

In the context of diabetes, epidemiological studies from helminth-endemic areas such as Indonesia, rural China, India and Aboriginal communities from North-West Australia found an inverse relationship between helminth infection and incidence of T2D (17–20). Additionally, it has been shown that infections of mice with different species of parasitic helminths are associated with significant increases in ILC2s, eosinophils, M2 MACs, and Th2 cytokines that result in restoration of glucose levels and improved insulin sensitivity in mouse models of obesity (3, 4, 21–24).

Diabetes has been found to associate with alterations in the composition of the gut microbiota. Helminth infection in humans has also been shown to modulate the composition of the gut microbiota (25–27). For example, celiac disease patients infected with the hookworm *Necator americanus* and challenged with gluten showed improved oral tolerance to gluten and displayed an increased species richness in intestinal microbial species, notably the Bacteroidetes (27–29). A protective role for helminth-microbiota interaction in mice has been demonstrated against many inflammatory diseases such as allergy (30), IBD (31) and obesity (32). Human studies as well as studies in animal models of obesity and T2D revealed a shift in the abundance of the dominant gut phyla Bacteroidetes and Firmicutes (7). Shifts in the abundance of these phyla has also been observed after infection with the gastrointestinal nematodes *Nippostrongylus brasiliensis*, *Trichuris muris*, and *Heligmosomoides polygyrus* (33–36), suggesting that helminth infections might have a positive role in maintaining gut homeostasis and preventing the development of T2D *via* modulation of the gut microbiota and short chain fatty acids (SCFAs) (32, 37).

Previous studies have focused on the prophylactic effects of gastrointestinal nematode infection on high-fat diet-induced metabolic syndrome. To better reflect the current pandemic of human T2D, we infected mice fed on both high-fat (HF) and high-glycaemic index (HGI) diets before and after the onset of metabolic syndrome. We showed that infection with *N. brasiliensis* maintains glucose homeostasis both prophylactically and therapeutically, probably *via* induction of Th2 immune responses in lymphoid and non-lymphoid tissues in mice. Infection with *N. brasiliensis* was also associated with changes in some phyla and orders of the gut microbiota.

## Materials and Methods

### Ethics Statement

All procedures were approved by the James Cook University Animal Ethics Committee, ethics application number A2244. The study protocols were in accordance with the 2007 Australian Code of Practice for the Care and Use of Animals for Scientific Purposes and the 2001 Queensland Animal Care and Protection Act. *N. brasiliensis* was maintained in Sprague–Dawley rats (Animal Resources Centre, Perth, WA, Australia) as described elsewhere (38) (Ethics application number A2300).

#### Animals and Diet

Male C57BL/6 wild-type (WT) (JCU Townsville) mice were used for all experiments (10 mice per group). At 5 weeks of age mice were divided into three groups: (i) normal chow (NC); High Glycaemic Index (HGI) diet with a glycaemic index of close to 100 (SF03-30; Speciality Feeds, Western Australia); High Fat (HF) diet where 61% of total energy is from lipids (SF07-066; Speciality Feeds, Western Australia). **Table S1** describes the composition of each diet.

### Helminth Infection

*N. brasiliensis* life cycle was maintained in our laboratory at James Cook University in a specified pathogen free environment. Briefly, feces from *N. brasiliensis*-infected rats were collected from days 5–9 post-infection. Egg-containing feces were mixed with an equal amount of water and charcoal, distributed into Petri dish plates and incubated at 26°C. One week after incubation, L3 were collected from the fecal/charcoal culture plate, washed three times with PBS, then all infections with *N. brasiliensis* were performed by inoculating subcutaneously 500 third-stage larvae of *N. brasiliensis* (*Nb*L3) into the skin over the interscapular region. *N. brasiliensis* is immunologically cleared from mice within a few weeks, so we reinoculated mice once every month with 500 *Nb*L3 starting at 6 weeks of age for mice receiving prophylactic infections or 24 weeks of age for mice receiving therapeutic infections.

### Fasting Blood Glucose and Oral Glucose Tolerance Test

Food was withdrawn for 6 h then fasting blood glucose (FBG) was measured in the unfed mice. Blood sampling was performed by tail bleeding. Mice were screened for blood glucose levels every 2 weeks using Accu-Check^®^ Performa (Roche). Mice were considered diabetic when glucose levels reached >12.0 mmol/L. For the oral glucose tolerance test (OGTT), after initial blood collection (time 0) in the 6-h unfed mice, mice were administered D-glucose orally (2 g/kg body weight) by gavage. Blood sampling was performed by tail bleeding at 15, 30, 60, 90, and 120 min after administration of glucose.

### Isolation of Mesenteric Lymph Nodes, Adipose Tissue, and Liver

In brief, epididymal fat pads or liver from male mice fed with NC, HGI, or HF diets were removed and minced into small pieces. Minced tissues were then transferred to a 50 ml conical tube containing 1 ml DPBS (0.5% BSA) (Sigma) and 3 ml collagenase type II (Life Technologies), and incubated in a rotating shaker (200 rpm) at 37°C for 35 min. The homogenates were filtered through a 70 μm tissue strainer into a new tube and centrifuged at 500 g for 10 min. at 4°C. Following centrifugation, the supernatant was discarded and the pellet was resuspended in 1× red blood cell lysis buffer (Sigma) followed by a washing step with 5 ml FACS buffer, and a final centrifugation at 500 g for 10 min at 4°C.

Mesenteric lymph nodes (MLN) were collected and transferred to a 5 ml tube containing 1 ml of RPMI media (Gibco), then filtered through a 70 μm tissue strainer. Cell viability was assessed by Trypan Blue and cells were blocked using FcR blocking reagent (BD biosciences) for FACS analysis.

### Flow Cytometry

Cell surface marker analysis was performed using flow cytometry. Single-cell suspensions prepared from MLN, adipose tissue (AT) and liver were collected from mice at the times indicated. Cell surface markers were stained for 30 min at 4°C with rat anti-mouse CD3/CD19-CF594 (Clone:145-2C11,1D3) F4/80-APC (Clone: T45-2342), CD11c-FITC (Clone: HL3), CD301-pecy7 (Clone: LOM-14), CD64-PerCp-Cy5.5 (Clone: X45-5/7.1), CD11b-BV650 (Clone: M1/70), Ly6G-efluor700 (Clone: 1A8) and Siglec-F-PE (Clone: E50-2440) (BD Bioscience). All antibody incubations were performed at 4°C for 30 min (isotype controls were included). Data were acquired using a BD FACS Aria and analyzed using FlowJo software (Tree Star, Inc).

### Quantitative Real-Time PCR

A small piece (<0.5 cm) of AT, liver and small intestine (SI) (jejunum) was collected in a 2 ml Eppendorf tube containing 1 ml TRIzol-reagent (Sigma) and homogenized using a TissueLyzer (QIAGEN). Tissues were homogenized and RNA was extracted using TRIzol-reagent (Sigma) following the manufacturer’s protocol. RNA samples were reverse transcribed to cDNA as follows. After RNA quantification, 50–70 ng of each sample was transferred to a 0.2-ml tube and 1 μl of each of oligo(dT) (Qiagen) and 10 mM dNTPs were added, followed by incubation at 65°C for 5 min in a Veriti 96-well thermal cycler (Applied Biosystems) followed by incubation on ice for 2 min. Four (4) μl of first strand buffer (Thermo Fischer), 1 μl of each of 0.1 M DTT (Thermo Fischer), RNAse out and 0.5 μl of Superscript III (Thermo Fischer) were added to the sample. The sample was incubated for 60 min at 55°C, then 15 min at 70°C. Finally, cDNA was quantified on a Nanodrop 2000 (Thermo Scientific).

For qPCR reactions, 100 ng of cDNA was mixed with 12.5 μl of SYBR Green and 2.5 μl of each primer of the selected genes in a total volume of 25 μl per sample. A Rotor-Gene Q (QIAGEN) was used for real time thermal cycling. All genes were normalized for levels of transcription relative to the housekeeping gene β-actin.

### Staining and Quantification of Eosinophils

A 1-cm piece of small intestine (SI) (jejunum) was fixed in 4% paraformaldehyde. The samples were processed in a Histocore Pearl automatic tissue processer, embedded in paraffin and cut in 5 µm sections with a rotary microtome. The slides were first dewaxed with Xylene (2–6) min, absolute ethanol for 2–6 min, 70% ethanol for 1 min and DI water for 1 min. Slides were then stained with Congo Red solution (Sigma) for 1 h as per the manufacturer’s instructions, followed by DI water for 2 min, Harris hematoxylin for 30 s, DI water for 2 min, Scotts tap water for 1 min, DI water for 1 min, 95% ethanol for 1–2 min, absolute ethanol for 1–2 min and xylene for 1–2 min. Cover slips were then placed on slides before scanning with an Aperio CS2 scanner (Leica). Quantification of eosinophils was performed by counting the eosinophils in 15 fields of view (magnification x40) in 2 sections per group.

### Data Analysis

Data were tested for statistical significance using GraphPad Prism software (version 8). A Mann-Whitney U test was applied to test statistically significant differences between two unpaired groups with non-parametric distribution. Data that were normally distributed were tested for statistical significance using the unpaired t test for comparisons of two groups or the ANOVA test followed by the Holm-Sidack multiple-comparison test to compare more than two groups. Values of p < 0.05 were considered statistically significant. Results are expressed as SEM or means ± SD. Significance values are indicated as *p < 0.05; **p < 0.01.

### DNA Extraction and Bacterial 16S rRNA Illumina Sequencing

After mice were sacrificed, jejunum samples were collected and stored immediately at −80°C for further analysis. DNA extraction and 16s rRNA sequencing were performed by the Australian Centre for Ecogenomics, University of Queensland, Brisbane. In brief, a total of 50 to 100 mg of tissue sample was disrupted mechanically using a Powerlyzer 24 at 2,000 *g* for 5 min. A QIAamp 96 PowerFaecal QIAcube HT Kit (Qiagen) was used to process the resulting lysate as per the manufacturer’s instructions, and a Qubit assay (Life Technologies) was used for measuring DNA concentration, which was then adjusted to a concentration of 5 ng/μl. The 16S rRNA gene was targeted, using the 803 forward primer (5′-TTAGAKACCCB NGTAGTC-3′) and 1392 reverse primer (5′- ACGGGCGGTGWGTRC-3′) to cover the V6-V8 regions. Preparation of the 16S library was performed following the protocol outlined in the Illumina guide. In the first stage, 466 bp of the PCR products were amplified. The resulting PCR amplicons were then purified using Agencourt AMPure XP beads (Beckman Coulter). The purified DNA was indexed with unique 8cbp barcodes using the Illumina Nextera XT 384 sample Index Kit A-D (Illumina FC-131-1002). The QIAquick Gel Extraction Kit (Qiagen) was used for the isolation of the indexed amplicons as per the manufacturer’s instructions for the specific band at 450 bp (running at 610 bp with the adaptor sequence). Then, the resulting purified indexed amplicons were pooled together in equimolar concentrations and sequenced on a MiSeq Sequencing System (Illumina) using paired end (2 x 300 bp) sequencing with V3 chemistry in the Australian Centre for Ecogenomics according to the manufacturer’s protocol. Passing quality control of resulting sequence was determined as 10,000 raw reads per sample prior to data processing and passing quality control metrics in line with Illumina supplied reagent metrics of overall Q30 for 600cbp reads of >70%.

### Bioinformatics and Statistical Analysis

Sequence data were analyzed using a modified version of MetaGalaxyDE (39). Briefly, raw reads were run through fastqc for quality control, Trimmomatic (40) for adapter trimming and low quality base removal, QIIME (41) for Operational Taxonomic Units (OTUs) generation, and BLAST (42) for OTU identification. Within QIIME, low-quality reads are filtered with all remaining sequences de-multiplexed and chimeric sequences removed using UCHIME (43). Sequences were subsequently clustered into OTUs on the basis of similarity to known bacterial sequences in the Greengenes database (44) (cut-off: 97% sequence similarity) using the UCLUST software (45).

For each biom file, the taxonomic observation and metadata was added using biom API (46) which was next loaded into the R package phyloseq (47). Within phyloseq, the DESeq2 (48) API was called and a list of most differentially expressed bacteria generated for all possible pairings of conditions (NC and NC infected with *N. brasiliensis*, T2D mice fed HGI and T2D mice fed HGI infected with *N. brasiliensis* or T2D mice fed HF and T2D mice fed HF infected with *N. brasiliensis*). All subsequent plots were generated using ggplot2 and Calypso online software (version 8.84) (http://cgenome.net/calypso/) (49). Within Calypso, data were normalized by total sum normalization (TSS) combined with square root transformation. Multivariate redundancy analysis to overall differences in the microbial profile between groups and Adonis based on the Bray-Curtis dissimilarity and spearman’ index was used. Differences in bacterial alpha diversity (Shannon diversity) and richness between groups were used. Values of p < 0.05 were considered statistically significant following false discovery rate (FDR) correction. Differences in the bacterial taxa abundance between groups were assessed using ANOVA-like differential expression analysis (ALDEx2) and quantitative visualization of phyla abundance.

### Short Chain Fatty Acid Analysis by NMR

Fresh fecal pellets were collected at different time points of the experiment and stored at −80°C for metabolite extraction and analysis. Once thawed, fecal pellets were mixed with 600 μl of PBS and stored at room temperature for 20 min before manual disruption with a pipette tip and vortexing and centrifugation at 15,000 *g* at 4°C for 10 min. The supernatant was then transferred into a new microfuge tube and 50 μl of deuterated water (Cambridge Isotope Laboratories, Inc., USA) and 10 μl of 4,4-dimethyl-4-silapentane-1-sulfonic acid (DSS) were added, with the latter being a chemical shift reference. The samples were centrifuged again at 15,000 *g* at 4°C for 10 min and 550 μl of each sample was transferred into a 5 mm NMR tube. NMR spectra were acquired on a Bruker Avance III 600 MHz spectrometer (Bruker, Karlsruhe, Germany) and recorded at 600 MHz at 298 K equipped with a cryoprobe, using automated data collection *via* IconNMR software and the standard Bruker ^1^H cpmgpr1d pulse sequence (128 scans) to collect one-dimensional spectra. The NMR spectra were referenced to DSS. The software Metaboanalyst (v 4.0) was used for metabolites analysis and we focused specifically on detecting and comparing fecal SCFAs. The SCFA peaks were identified based on standard samples. The chemical shift region containing the water resonance (δ 4.68–4.89) was removed from the analysis. The calculated regions were normalized to the total sum and Pareto scaling for overall concentration differences prior to statistical data analysis with t-tests.

## Results

### Infection With *N. brasiliensis* Maintained Glucose Homeostasis and Reduced Body Weight Gain

**Figure 1:**
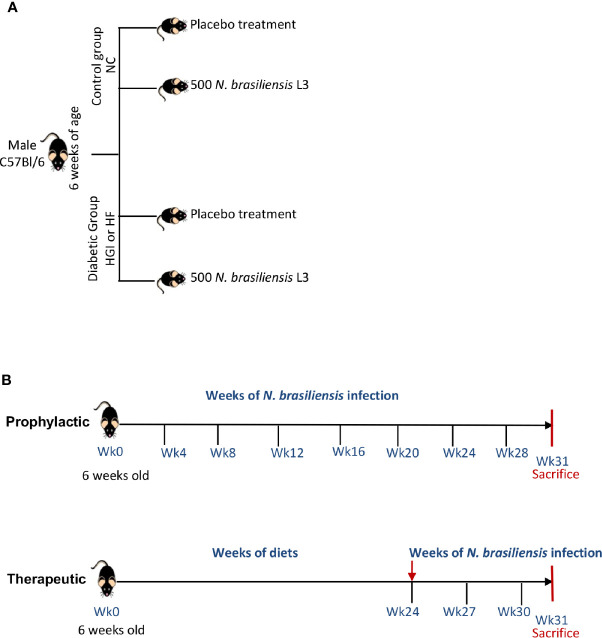
Experimental design **(A)** and timeline for infection of mice with *Nippostrongylus brasiliensis* third stage larvae (L3) **(B)**. NC, Normal Control diet; HGI, High Glycemic Index diet; HF, High Fat diet.

In order to address the prophylactic and therapeutic effects of infection with *N. brasiliensis* on the outcome of T2D, we used two different models of diet (HGI and HF) to induce T2D in C57BL/6 mice. At 6 weeks of age, male C57BL/6 mice were either kept on a normal control diet (NC) or fed either HGI or HF diets for up to 31 weeks to induce T2D (**Figure 1A**). To ascertain the prophylactic effect of the infection on T2D, mice were infected at week 6 and re-infected once every month until the end of the experiment (week 31) to ensure continuous parasite infection. To ascertain the therapeutic effect, infection with *N. brasiliensis* started at week 24 and continued once every 3 weeks for a total of three infections until the end of the experiment (week 31) (**Figure 1B**).

**Figure 2:**
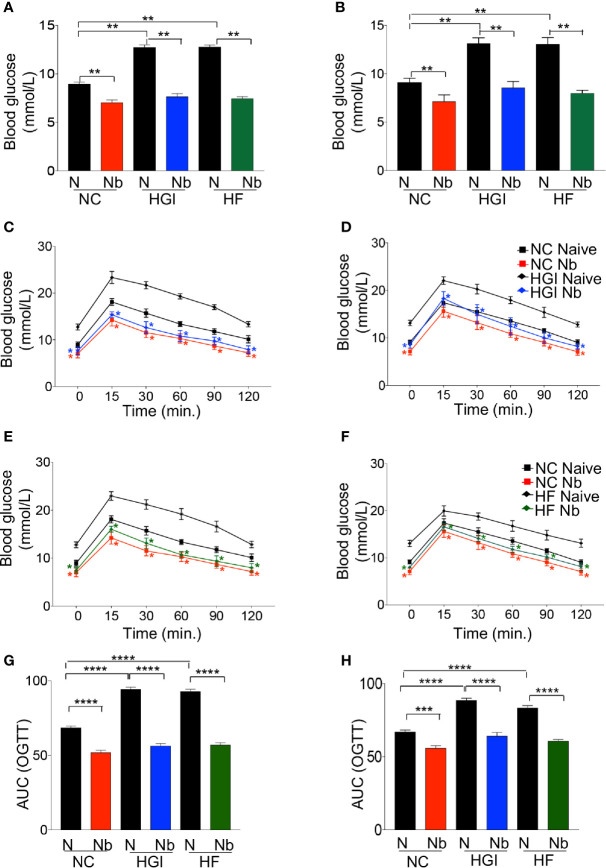
*Nippostrongylus brasiliensis* infection decreased fasting blood glucose (FBG) and improved glucose metabolism in high glycaemic index (HGI) and high fat (HF) diet models of type 2 diabetes. C57BL/6 mice were fed normal control (NC), HF or HGI diet and infected once monthly with 500 infective larvae of *N. brasiliensis* commencing at 6 weeks of age for prophylactic infections and 24 weeks of age for therapeutic infections. **(A)** FBG in mice fed on different diets and administered prophylactic infection with *N. brasiliensis*. **(B)** FBG in mice fed on different diets and administered therapeutic infection with *N. brasiliensis*. Oral glucose tolerance test (OGTT) in mice fed on NC or HGI diets and administered prophylactic **(C)** or therapeutic **(D)** infection with *N. brasiliensis*. Oral glucose tolerance test (OGTT) in mice fed on NC or HF diets and administered prophylactic **(E)** or therapeutic **(F)** infection with *N. brasiliensis*. Area under the curve (AUC) in mice fed on different diets and administered prophylactic **(G)** or therapeutic **(H)** infection with *N. brasiliensis*. Statistical significance was determined with Student’s t test or Two-way analysis of variance (ANOVA). Data are expressed as means ± SEM or means ± SD are representative of two experiments where n = 5/group. *p < 0.05; **p < 0.01; ***p < 0.001; ****p < 0.0001.

As predicted, mice on either HGI or HF diets had a significant increase in the level of FBG compared to those on NC diet (**Figures 2A, B**). Prophylactic infection as well as therapeutic infection with *N. brasiliensis* significantly decreased the FBG levels in the diabetic groups fed on HGI and HF diets, compared to their respective uninfected groups fed on othose same diets (**Figures 2A, B**). A significant reduction in FBG was also noted in the NC group infected with *N. brasiliensis* when compared with their uninfected littermates, both prophylactically and therapeutically. A similar result was also observed for the OGTT test. HGI and HF diet infected mice had significantly lower levels of blood glucose than their respective control (uninfected) groups at all time points, both prophylactically and therapeutically (**Figures 2C–F**). Moreover, the NC group infected with *N. brasiliensis* had significantly lower levels of blood glucose than their respective control at all time points. Of note, the blood glucose levels of the HGI and HF diet infected groups were also comparable to those mice on a NC diet that were infected with *N. brasiliensis* (**Figures 2C–F**).

### *N. brasiliensis* Infection Slowed Weight Gain in HGI and HF Diet Models of T2D

**Figure 3:**
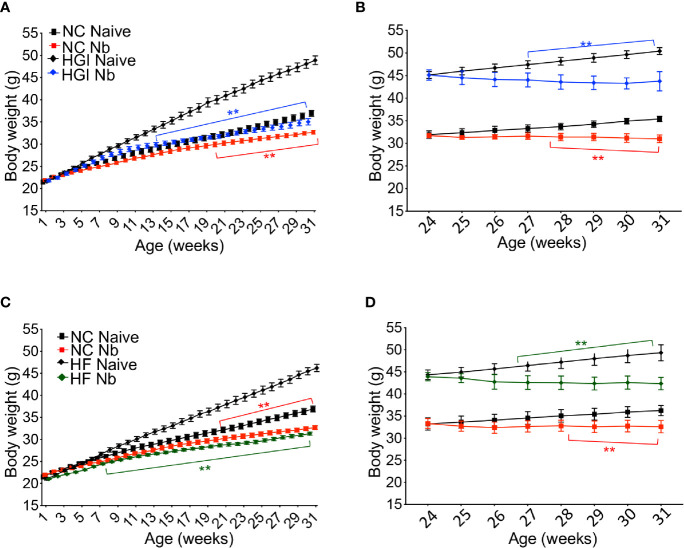
*Nippostrongylus brasiliensis* infection reduced weight gain in high glycemic index (HGI) and high fat (HF) diet models of T2D. C57BL/6 mice were fed normal control (NC), HF, or HGI diet and infected once monthly with 500 infective larvae of *N. brasiliensis* commencing at 6 weeks of age for prophylactic infections and 24 weeks of age for therapeutic infections. **(A)** Body weight of mice fed on HGI diet and administered prophylactic infection with *N. brasiliensis* (Nb). **(B)** Body weight of mice fed on HGI diet and administered therapeutic infection with Nb. **(C)** Body weight of mice fed on HF diet and administered prophylactic infection with Nb. **(D)** Body weight of mice fed on HF diet and administered therapeutic infection with Nb. Statistical significance was determined with Two-way analysis of variance (ANOVA). Data are expressed as mean ± SD and are representative of two experiments where n = 5/group. *p < 0.05; **p < 0.01.

Reduction in the rate of body weight gain was also observed as a result of infection. As expected, mice on either HGI or HF diets gained significantly more weight compared to mice on a NC diet (**Figure 3**); however, either prophylactic (**Figures 3A, C**) or therapeutic (**Figures 3B, D**) infection with *N. brasiliensis* significantly reduced the body weight gain in the diabetic groups fed on HGI and HF diets compared to their uninfected counterparts. Of note, significant reduction in body weight was also observed in the NC diet group infected with *N. brasiliensis* compared with the uninfected NC group, both prophylactically and therapeutically. When food weight was calculated on a weekly basis for each cage, no differences in food consumption were detected at any time between these two groups (data not shown).

These data indicate that in response to *N. brasiliensis* infection, HGI and HF diet groups maintained low levels of FBG and displayed improved glucose metabolism compared to uninfected controls.

### *N. brasiliensis* Infection Induces Local Eosinophilia and Th2 Immune Responses

**Figure 4:**
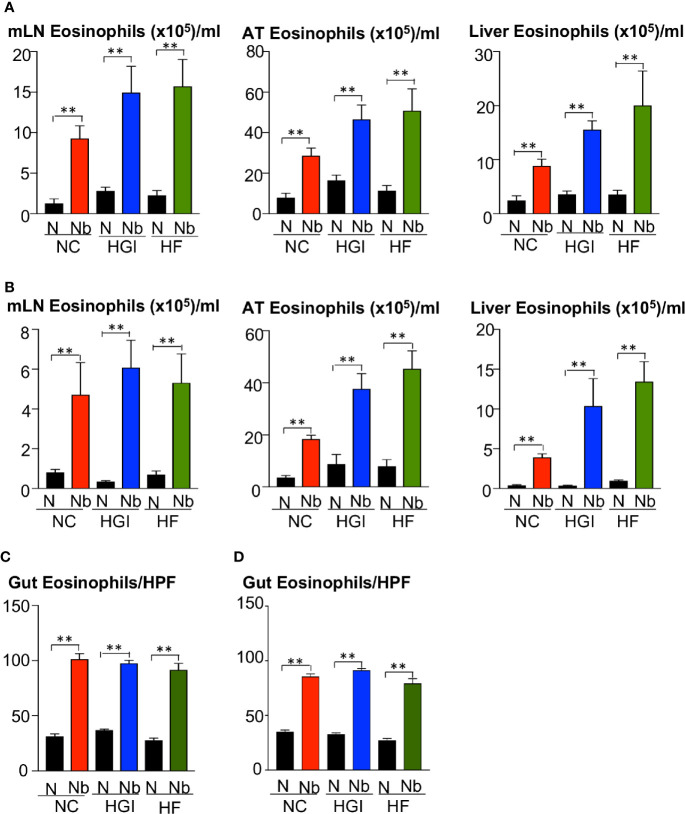
Increase in the frequency of eosinophils in the mesenteric lymph nodes (MLN), adipose tissue (AT), liver, and duodenum in mice fed on different diets and infected or not with *Nippostrongylus brasiliensis*. C57BL/6 mice were fed normal control (NC), high fat (HF), or high glycemic index (HGI) diet and infected once monthly with 500 N*. brasiliensis* infective larvae from 6 weeks of age [prophylactic, panel **(A)**] or 24 weeks of age [therapeutic, panel **(B)**]. Eosinophil frequency and total numbers in MLN, AT, and liver are shown. Eosinophil numbers per high power field (HPF) (magnification x40) in the gut are shown in panel **(C)** (prophylactic) and panel **(D)** (therapeutic). Statistical significance was determined with Student’s t test. Data are expressed as mean ± SEM and are representative of two experiments where n = 5/group. **p < 0.01.

**Figure 5:**
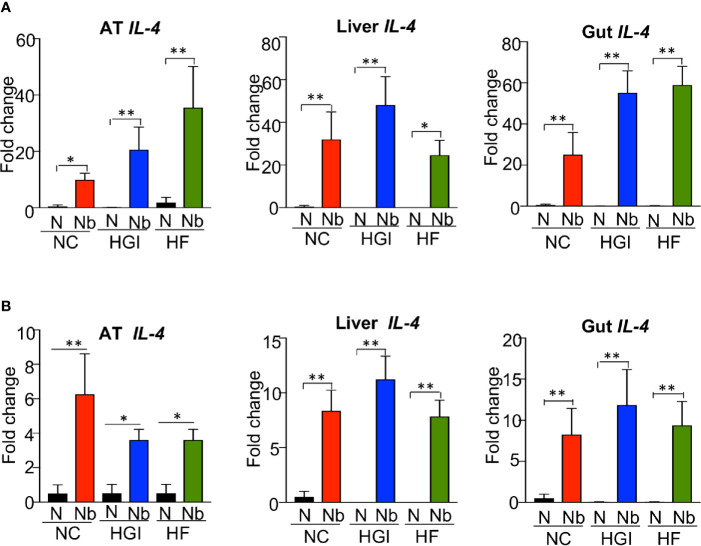
Increased expression of *Il4* in adipose tissue (AT), liver and gut of mice fed on different diets and infected with *Nippostrongylus brasiliensis* compared to uninfected mice. C57BL/6 mice were fed normal control (NC), high fat (HF), or high glycemic index (HGI) diet and infected once monthly with 500 N*. brasiliensis* infective larvae from 6 weeks of age [prophylactic, panel **(A)**] or 24 weeks of age [therapeutic, panel **(B)**]. Statistical significance was determined with Student’s t test. Data are expressed as mean ± SEM and are representative of two experiments where n = 5/group. *p < 0.05; **p < 0.01.

**Figure 6:**
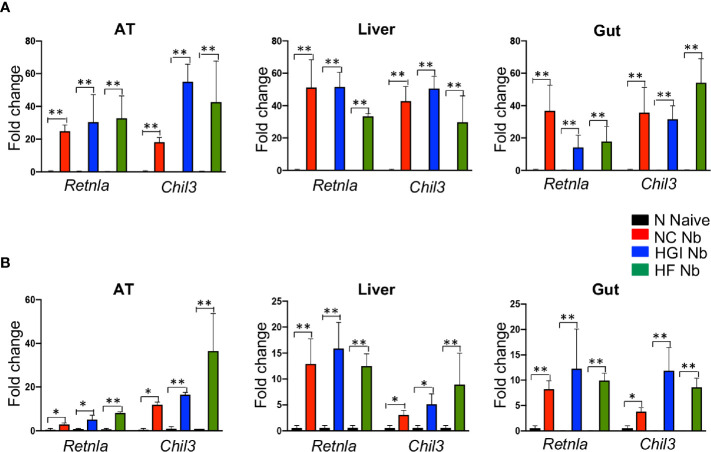
Increased expression of *Retnla* and *Chil3* genes in adipose tissue (AT), liver, and duodenum of mice fed on different diets and infected with *Nippostrongylus brasiliensis* compared to uninfected mice. C57BL/6 mice were fed normal control (NC), high fat (HF), or high glycemic index (HGI) diet and infected once monthly with 500 N*. brasiliensis* infective larvae from 6 weeks of age [prophylactic, panel **(A)**] or 24 weeks of age [therapeutic, panel **(B)**]. Data are expressed as mean ± SEM and are representative of two experiments where n = 5/group. *p < 0.05; **p < 0.01.

To determine whether *N. brasiliensis* infection induced a potent Th2 cytokine response accompanied by eosinophilia and alternative activation of MACs, mice fed a NC, HGI or HF diet were infected with 500 N*. brasiliensis* L3 and sacrificed at week 31 of the experiment (**Figure 1B**). qPCR analysis was performed on AT, liver and SI to assess M2 MAC expression markers. In response to both prophylactic and therapeutic *N. brasiliensis* infection, there was a significant increase in the total number of eosinophils in the MLN, AT and liver of the diabetic groups as well as the NC group compared to their uninfected littermates (**Figures 4A, B**). Moreover, quantification of eosinophil numbers in the gut revealed a significant increase of these cells in the NC, HGI and HF diet groups infected with *N. brasiliensis* compared to their respective uninfected groups (**Figures 4C, D**). Both prophylactic and therapeutic *N. brasiliensis* infection significantly upregulated expression of genes encoding major Th2 cytokines and associated MAC proteins in all tissues assessed (**Figure 5**). In response to infection, elevated expression of *IL-4* was detected in the AT, liver and SI of mice on all three diets compared to their respective uninfected groups (**Figures 5A, B**). Infection also resulted in increased expression of *Retnla* (encoding the resistin-like alpha protein) and *Chil3* (chitinase-like protein 3) markers of M2 macrophages and a Th2 environment in these same tissues (**Figures 6A, B**).

### Infection With *N. brasiliensis* Resulted in Altered Alpha Diversity and Microbial Richness in Mice Fed on NC, HGI, and HF Diets

**Figure 7:**
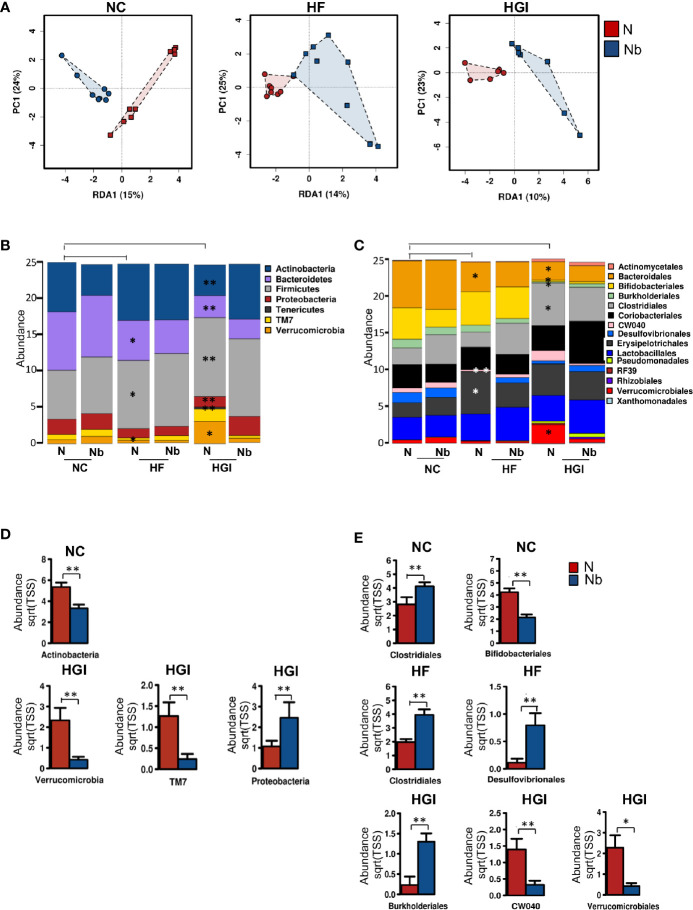
Multivariate analysis of differences in the microbial profiles in the small intestine of *Nippostrongylus brasiliensis* (Nb) infected and uninfected (naïve, N) C57BL/6 mice fed on normal control (NC), high fat (HF), or high glycaemic index (HGI) diet **(A)**. Relative abundance of bacterial phyla in the small intestine of Nb and N mice fed on NC, HF, or HGI diet **(B)** Relative abundance of bacterial orders in the small intestine of Nb and N mice fed on NC, HF, or HGI diets **(C)**, and abundance of defined taxa where significant differences between infected and uninfected groups were detected **(D, E)**. Mice were infected once monthly from 6 weeks of age with Nb infective larvae. P values are based on ANOVA-like differential expression analysis and are representative of two experiments where n = 5/group. *p < 0.05; **p < 0.01.

We wanted to address the effect of infection with *N. brasiliensis* on the composition of the gut microbiota in mice fed NC, HGI and HF diets. SI samples were collected at termination (week 31) to determine the differences in the composition of gut microbiota between infected and uninfected groups. Multivariate redundancy analysis on OTU level showed a different clustering in the microbial profiles of the infected groups compared to the uninfected control groups for all diets (NC, HGI and HF) (**Figure 7A**). Adonis analysis revealed significant differences between infected and uninfected groups on all diets using at least one of the two indices (Bray-Curtis or spearman) (**Supplementary Table 2**). No significant differences in the Shannon index and species richness were observed in *N. brasiliensis* infected groups on all three diets compared to their respective uninfected groups (**Figure S1**).

In general differences were detected in the abundance of some bacterial taxa between uninfected groups fed on diabetic diets (HF or HGI) and those fed NC diet, as well as between infected and uninfected groups fed on all diets used in this study (**Figures 7B–E**). At the phylum level, mice fed either HF or HGI diets had a significant decrease in the abundance of Bacteroidetes and significant increase in Firmicutes compared to the NC group (**Figure 7B**). However, infection status had no impact on the abundance of these phyla (**Figure 7B**). Moreover, Actinobacteria was significantly decreased in the uninfected HGI group compared to the uninfected NC group, however no such changes were detected between the HF and NC groups (**Figure 7B**). On the other hand, the NC group infected with *N. brasiliensis* had a significant decrease in the abundance of Actinobacteria compared to their uninfected littermates (**Figure 7D**). Also the abundance of Actinobacteria was not different between HF infected and uninfected groups (**Figure 7B**). The uninfected HF diet group displayed decreased abundance of TM7 and Verrucomicrobia compared to the uninfected NC group, whereas the uninfected HGI group displayed increased abundance of both phyla when compared to the uninfected NC group (**Figure 7B**). On the other hand, a significant increase in the abundance of Proteobacteria and a significant decrease in the abundance Verrucomicrobia and TM7 were detected in the infected HGI group compared to the uninfected HGI group (**Figure 7D**). However these changes were not detected between infected and uninfected NC or HF groups (**Figure 7B**). No significant differences were detected in any phyla between infected and uninfected groups fed HF diet (**Figure 7B**).

At the order level, both diabetic groups showed significantly lower abundance of Bacteroidales (Bacteroidetes phylum) compared to the NC group (**Figure 7C**). However, no differences were detected in any of the three diet groups infected with *N. brasiliensis* compared to their uninfected naïve groups (**Figure 7C**). As a result of the diets, significant decrease in the abundance of Bifidobacteriales (Actinobacteria phylum) and Burkholderiales (Proteobacteria phylum) were detected in the uninfected HGI group, but not in the uninfected HF group when compared with the NC group (**Figure 7C**). On the other hand, the NC group infected with *N. brasiliensis* showed a significant decrease in the abundance of Bifidobacteriales (but not Burkholderiales) compared to the uninfected naïve group (**Figures 7C, E**). However, HGI mice infected with *N. brasiliensis* had significantly higher abundance of Burkholderiales and showed no changes in the abundance of Bifidobacteriales when compared to their uninfected naïve group (**Figures 7C, E**). HF mice that were infected with *N. brasiliensis* showed no differences in the abundance of both Bifidobacteriales and Burkholderiales compared to their uninfected group (**Figure 7C**). Moreover, the uninfected HGI group had significantly higher abundance of Clostridiales (Firmicutes phylum) compared to the NC group, but no changes were detected between HF and NC groups (**Figure 7C**). *N. brasiliensis* infection significantly increased the abundance of Clostridiales in both the NC and HF groups infected with *N. brasiliensis* compared to their respective uninfected diet-matched groups (**Figure 7E**). Furthermore, the abundance of both the CW040 order (TM7 phylum) and Desulfovibrionales (Proteobacteria phylum) was significantly decreased in the HF diet group compared to the NC diet group. While those on the HGI diet had increase in the abundance of CW040 and decrease in the abundance of Desulfovibrionales compared to the NC diet, but this was not significant (**Figure 7C**). *N. brasiliensis* infection caused a significant elevation in the abundance of Desulfovibrionales and trend toward increased abundance of CW040 in the HF diet group infected with *N. brasiliensis* compared to their uninfected group (**Figure 7E**). *N. brasiliensis* infection significantly decreased the abundance of CW040 and showed a trend toward increased abundance of Desulfovibrionales in the infected HGI group compared to their uninfected diet-matched group (**Figures 7C, E**). As a result of the diets, the abundance of Verrucomicrobiales order (Verrucomicrobia phylum) was significantly increased in the HGI but not HF groups compared to the NC group (**Figure 7C**). On the other hand, the abundance of the Verrucomicrobiales was significantly decreased in the infected HGI group compared to the uninfected group (**Figure 7E**).

To summarize, the impact of *N. brasiliensis* infection on the microbiome at the phylum level was most notable in mice on the HGI diet, with reduced abundance of Verrucomicrobia and TM7 phyla in infected mice and increased abundance of Proteobacteria in HGI infected mice. At the order level, infection with *N. brasiliensis* resulted in increased abundance of Clostridiales and Desufovibrionales in mice on the HF diet and Burkholderiales in mice on the HGI diet.

### Infection With *N. brasiliensis* Alters Fecal Short Chain Fatty Acid Content in Mice Fed on Different Diets

Metabolic profiling of fecal extracts from infected and uninfected mice fed on the three different diets was carried out using NMR spectroscopy. We compared the SCFAs acetate, butyrate and propionate between the uninfected and *N. brasiliensis* infected groups by t-test. For each diet at least one of the SCFAs was present in higher quantities (p < 0.05) in infected versus uninfected mice (**Table 1**; **Figure S2**).

**Table 1:** Effect of *Nippostrongylus brasiliensis* on fecal short chain fatty acid levels in mice fed different diets.

Diet	SCFA	p-value	Change upon infection
Normal			
	Acetate	ns	
	Butyrate	0.04	increase
	Propionate	0.03	increase
High glycemic index			
	Acetate	ns	
	Butyrate	0.007	increase
	Propionate	ns	
High fat			
	Acetate	ns	
	Butyrate	ns	
	Propionate	0.046	increase

## Discussion

Diabetes is recognized as the world’s fastest growing chronic condition (1). Helminth infections have been associated with a lower prevalence of T2D due to their ability to induce type 2 immune responses (17–20). We therefore set out to investigate the role of helminth-induced type 2 immunity and the potential mechanisms underlying protection against the development of T2D-induced insulin resistance. C57BL/6 mice were fed a HGI or HF diet and infected frequently with *N. brasiliensis*. This strain of mice is genetically susceptible to obesity, glucose intolerance, hyperglycaemia and T2D when fed a HF or HGI diet (50, 51). We demonstrated that infection with *N. brasiliensis* had a beneficial effect, both prophylactically and therapeutically against T2D in two different diabetes-inducing diets. Our findings are consistent with a role for helminth infection in promoting type 2 immune responses by eliciting eosinophil accumulation in MLN, AT, liver and SI, with increased expression of genes encoding for key Th2 cytokines and M2 MACs in AT, liver and SI. We did not quantify levels of regulatory cells and cytokines, such as regulatory T cells, IL-10 and TGF-β, but a role for such a response in protecting against T2D in this model is plausible and should be addressed in the future.

In line with our results, a number of studies have shown improved glucose tolerance in mouse models of diabetes induced by a range of parasitic nematodes with distinct tissue niches. For example, studies utilizing the HF diet model of obesity showed improvements in glucose tolerance of obese mice after infection with the filarial nematode *Litomosoides sigmodontis* or administration of soluble adult worm extract (21). Infection with *H. polygyrus* also resulted in decreased body weight gain and improved glucose and lipid metabolism, and an associated increase in Th2/Treg immune responses in the MLNs, AT and SI. Moreover, infected mice on a HF diet displayed dysregulated expression of genes and proteins involved in energy expenditure and lipid metabolism in AT and liver (23, 24). Infection with parasitic platyhelminth flatworms (distinct phylum from the Nematoda) has also been shown to protect against metabolic syndrome. Chronic *Schistosoma mansoni* infection and administration of schistosome soluble egg antigens resulted in increased numbers of AT eosinophils, M2 MACs and Th2 cytokines, and a corresponding decrease in body weight gain and improved insulin sensitivity in obese mice (52).

Recently, eosinophils in particular have been implicated in glucose homeostasis and energy expenditure. These cells play an unexpected role in metabolic homeostasis through maintenance of adipose M2 MACs. Absence of eosinophils resulted in increased body weight gain and impaired glucose tolerance in mice (3, 4, 53). Moreover, in the absence of eosinophils, mice exhibit a defect in lipid metabolism in the liver and SI, an increase in the expression of pro-inflammatory IFN-γ and a decrease in the expression of IL-4 and IL-13 in AT (54).

In our work, *N. brasiliensis* induced MLN, AT, liver and SI eosinophilia, and increased gene expression of M2 MAC markers in AT, liver and SI. This was consistent with other studies which showed that infection with *N. brasiliensis* induced adipose eosinophilia and M2 MACs, enhanced glucose tolerance and lipid metabolism and ameliorated body weight gain in different mouse models of obesity (3, 4, 22). These studies have proposed mechanisms by which these cells might influence AT homeostasis. Eosinophils in bone marrow and their recruitment into white AT are largely controlled by IL-5. Mechanistically, the increase in eosinophil numbers in our mice fed a HGI or HF diet following *N. brasiliensis* infection may be the result of local and systemic increases in eosinophils and Th2 cytokines, as well as increases in M2 MAC numbers that regulate many key events involved in the control of metabolic homeostasis. It is not yet clear whether the eosinophil-mediated regulation of obesity-induced insulin resistance and AT inflammation is due to the direct and primary effects of eosinophils on insulin resistance or due to secondary effects of eosinophils on changes in body weight and adiposity. Further studies are required to elucidate the functions of helminth-induced eosinophils in terms of their beneficial and detrimental effects in driving metabolic reprogramming, and the therapeutic utility of this phenomenon for treating the global epidemic of metabolic disorders. On the other hand, changes in environmental and behavioral factors, such as diet, can modulate bacterial composition and metabolic activity (55), which can trigger an inflammatory immune response leading to the development of T2D (7, 55). Indeed, there is growing evidence that helminth infection alters the composition of the gut microbial community, conferring protection against immune mediated diseases such as allergic inflammation (30), IBD (31), and obesity (32). We therefore aimed to address the effect of infection with *N. brasiliensis* on the composition of the gut microbiota in mice fed on different diets. No significant changes were observed in α-diversity in response to *N. brasiliensis* infection in all diets studied (NC, HF and HGI); however, we still found a significant shift in the microbiota composition at the community level. This was in agreement with other studies with *N. brasiliensis* and *Hymenolepis diminuta* that also found no significant differences in α-diversity between infected and uninfected groups (33, 56), and suggests that inter-individual variation occurs in the microbiota composition as a result of infection (33).

In our study, *N. brasiliensis* infection resulted in a decrease in the abundance of Bifidobacteriales on the NC diet. In agreement with our findings, in human studies individuals with different helminth infections (i.e. *Trichuris* spp., *Ascaris* spp. and hookworm) had lower abundance of the Bifidobacteriales compared to uninfected individuals (26). Many linked the latter group with health benefits in T2D (57); however, others have reported them to cause infections (58). In HF diet induced obesity in rats, administration of four *Bifidobacteria* strains had different responses on energy and fat metabolism and showed no differences on serum insulin and glucose levels (59). Moreover, administration of *Bifidobacterium breve* to preterm infants, increased weight gain (60).

We found an increase in the abundance of Clostridiales in the NC and HF diet groups infected with *N. brasiliensis*. Infection with *H. polygyrus* attenuated allergic airway inflammation in mice inoculated with house dust mite allergen, which was associated with an increase in the abundance of Clostridiales (30). An increase in the abundance of Clostridiales was also reported after infection with *T. muris* which protected against colitis in *NOD2^-/-^* deficient mice *via* a mechanism involving type 2 immunity (31). Moreover, oral administration of a mixture of Clostridia strains known to induce CD4^+^ Foxp3^+^Tregs cells attenuated experimental colitis and allergic diarrhea (61, 62). It has also been reported that reduction in the abundance of Clostridia was associated with T2D in humans (63–66), and an increase in this group was associated with an improvement in glucose and lipid metabolism (67). Moreover, in two separate studies, oral administration of probiotic *Clostridium butyricum* also improved diabetic markers (fasting glucose, glucose tolerance, insulin tolerance, glucagonlike peptide and insulin secretion), decreased blood and liver lipids and restored colonic homeostasis of treated groups in two different models of T2D in mice (HF diet and leptin*^db/db^*) (68, 69). Of note, Clostridiales are abundant producers of the SCFAs that regulate colonic Treg cell homeostasis and strongly involved in the maintenance of overall gut function (70, 71).

In response to *N. brasiliensis* infection we also found a significant increase in the abundance of Desulfovibrionales and Burkholderiales (Proteobacteria) in the HF and HGI diet groups, respectively. In one study, the abundance of Proteobacteria also increased in *H. polygyrus*-infected mice fed a NC or a HF diet compared to naïve littermates (72). This phylum is, in part, responsible for regulating weight gain in HF diet fed mice (72). Abundance of the Desulfovibrionales was increased in mouse fecal samples as a result of infection with *Schistosoma haematobium* (73), *Ascaris lumbricoides*, and *Trichuris trichiura* (74). Moreover, the abundance of Desulfovibrionales and Burkholderiales was lower in obese mice (75). Cold exposure attenuated diet-induced obesity in mice, which was associated with an increase in the abundance of *Desulfovibrionaceae* (76). Desulfovibrionales are sulfate-reducing bacteria that use hydrogen or other compounds such as lactate, pyruvate and ethanol as electron donors to produce hydrogen sulfide (H_2_S) (77). H_2_S has been found to improve insulin secretion, improve glucose tolerance and reduce food intake *via* direct stimulation of glucagon-like peptide-1 (GLP-1) secretion in gut L-cells and indirectly *via* treatment with prebiotic chondroitin sulfate that enhanced the level of *Desulfovibrio piger* in the feces and colon of the treated group (78).

Levels of Verrucomicrobiales (Verrucomicrobia phylum) and CW040 (TM7 phylum) were significantly lower in the HGI group infected with *N. brasiliensis* in comparison with HGI-fed uninfected littermates. In agreement with our data, Verrucomicrobia were enriched in mice fed both HF and high sugar diets, but was not detected in mice fed NC diet (79). In another study, the abundance of Verrucomicrobia was significantly elevated when mice switched from NC to high sugar diet (80). Moreover, mice with leptin deficiency (db/db)-induced T2D showed an increase in the abundance of Verrucomicrobia (81). However, these mice exhibited a decrease in the abundance of Verrucomicrobia when subjected to an intermittent fasting regime which protected against diabetic retinopathy (82). The TM7 phylum is a recently identified bacterial group, composed of uncultivable and highly ubiquitous bacteria (83) and has been associated with inflammatory mucosal diseases, periodontitis, IBD and vaginosis in humans (84–86).

Many factors may play a role in modulating the abundance of microbial species in the gut. The variability in the microbiota community composition among different diets and as a result of infection might be due to differences in dietary substrates. Different microbial species might drive different effects on energy recruitment pathways. We also should consider the pathways for the metabolism of these substrates as well as the inter-individual variation in metabolism and its implications on the abundance of different microbial species in the gut (87).

Acetate, propionate and butyrate are the major SCFAs known to play important roles in gastrointestinal physiology and maintenance of gut integrity, metabolism and immune homeostasis (88). A complex interplay of multiple factors including, diet, gut microbiota, gut environment (eg. pH, and gas concentrations) can affect the formation of SCFAs and determine the amounts and types of SCFA that are produced (89). Changes in the concentrations of SCFAs have been implicated in modulating inflammatory pathology in distinct tissues in diseases such as IBD, cancer and T2D (88).

Interestingly, in addition to the impact of helminth infection in modifying host microbiome, several studies have also reported shifts in metabolites during helminth infection (90). We found that fecal SCFA levels were significantly elevated in *N. brasiliensis*-infected mice compared to uninfected mice for all diets tested, and this may have had a therapeutic benefit in modulating inflammation and suppressing insulin resistance.

Many studies have highlighted the roles of SCFAs in the regulation of appetite, weight gain, glucose and lipid metabolism (91). For instance, in overweight and obese individuals, colonic administration of SCFAs increased fat oxidation, energy expenditure and circulating levels of the satiety-stimulating hormones peptide YY (PYY) and GLP-1 concentration (92). Propionate administration stimulated the release of PYY and GLP-1 from colonic cells and increased their concentration in the circulation, reduced energy intake, intra-abdominal adipose tissue distribution, intrahepatocellular lipid content and prevented weight gain (93, 94). In mouse studies, administration of butyrate improved lipid and glucose metabolism which prevented HFD-induced obesity, insulin resistance, hypertriglyceridemia and hepatic steatosis. The effect was due to an increase in peroxisome proliferator–activated receptor-γ coactivator-1α expression that increased mitochondrial function and biogenesis in skeletal muscle and AT (95). The role of butyrate in regulation of the immune response has also been highlighted. Butyrate can regulate intestinal macrophage function to decrease the production of proinflammatory mediators such as nitric oxide (NO), IL-6 and IL-12 (96). Butyrate and propionate attenuated the activation of nuclear factor κB by LPS- stimulated neutrophils and inhibited the production of proinflammatory cytokines and NO (97). Butyrate and propionate have also bene shown to increase the numbers of Treg cells expressing Foxp3 in the colon, spleen and lymph nodes (98, 99).

Correlations between helminth presence and changes in the microbial composition have already been mentioned elsewhere throughout, but it is pertinent to note that many studies reported changes in the composition of the gut microbiota as a result of helminth infection and subsequent improvement in the outcome of immune mediated diseases. Whether the microbial composition changes we found in our study are a direct effect of helminth infection or a consequence of the host’s immune response to the infection, and whether these changes are essential to confer protection against T2D are yet to be investigated. A deeper understanding of the interplay between the host-microbiota-helminth triad and other variables may represent a new therapeutic strategy to prevent or even reverse the pathological effects of T2D.

## Data Availability Statement

The data sets presented in this study can be found in online repositories. The names of the repository/repositories and accession number(s) can be found below: NCBI, BioProject https://www.ncbi.nlm.nih.gov/bioproject/PRJNA676176.

## Ethics Statement

The animal study was reviewed and approved by James Cook University Animal Ethics Committee.

## Author Contributions

ZK, PG, JS, and AL conceived the project and designed the experiments. ZK performed data analysis and wrote original drafts of the manuscript. ZK and RA performed the experiments. RE provided material. AK provided advice with flow cytometry. MF and LK provided bioinformatics assistance and support. JW prepared histological sections. DW and ND provided assistance and support with NMR metabolomic analysis. ZK drafted the manuscript and all authors provided intellectual and editorial feedback. All authors contributed to the article and approved the submitted version.

## Funding

This work was supported by the National Health and Medical Research Council (NHMRC) through a program grant (1132975) and senior principal research fellowship (1117504) to AL, an AITHM Capacity Building grant to PG, AL, and MF, and an Australian Research Council Special Research Initiative award to the Australian Institute of Tropical Health and Medicine at James Cook University (SRI40200003). The funders had no role in study design, data collection and analysis, decision to publish, or preparation of the manuscript.

## Conflict of Interest

Author LK was employed by company Microba Pty Ltd.

The remaining authors declare that the research was conducted in the absence of any commercial or financial relationships that could be construed as a potential conflict of interest.

## Supplementary Material

Supplementary Figure 1 Shannon index and richness in the small intestine of Nippostrongylus brasiliensis (Nb) infected and uninfected (naïve, N) C57BL/6 mice fed on normal control (NC), high fat (HF) or high glycaemic index (HGI) diet. Mice were infected once monthly from 6 weeks of age with Nb infective larvae. P values are based on multiple linear regression and are representative of 2 experiments where n = 5/group.

Supplementary Figure 2 Example 1H NMR spectra of acetate, propionate and butyrate faecal extract used in this study.
