# Supplementary material for: Gastrointestinal Helminth Infection Improves Insulin Sensitivity, Decreases Systemic Inflammation, and Alters the Composition of Gut Microbiota in Distinct Mouse Models of Type 2 Diabetes
Source: Front Endocrinol (Lausanne). 2021 Feb 5;11:606530. doi: 10.3389/fendo.2020.606530 (PMC7892786; doi:10.3389/fendo.2020.606530)
Supplement: Supplementary file 1 [file DataSheet_1.docx]

| **NC diet** | **HF diet** | **HGI diet** |
| --- | --- | --- |
| barley | Casein (Acid) 200 g/Kg | Casein (Acid) 200 g/Kg |
| Lupins | Sucrose 100 g/Kg | Dextrose 505 g/Kg |
| Soya meal | Ghee (Butter fat) 8 g/Kg | Canola Oil 50 g/Kg |
| Fish meal | Cocoa Butter 336 g/Kg | Cocoa Butter 50 g/Kg |
| Mixed vegetable oils | Hydrogenated Vegetable Oil 15 g/Kg | Hydrogenated Vegetable Oil (Copha) 131 g/Kg |
| Canola oil | Cellulose 100 g/Kg | Cellulose 20 g/Kg |
| Wheat | Wheat Starch 67 g/Kg |  |
| Salt | Dextrinised Starch 132 g/Kg |  |
| Dicalcium phosphate | DL Methionine 3.0 g/Kg | DL Methionine 3.0 g/Kg |
| Calcium carbonate | Calcium Carbonate 13.1 g/Kg | Calcium Carbonate 13.1 g/Kg |
| Magnesium oxide | Sodium Chloride 2.6 g/Kg | Sodium Chloride 2.6 g/Kg |
| A Vitamin | AIN93 Trace Minerals 1.4 g/Kg | AIN93 Trace Minerals 1.4 g/Kg |
| Trace mineral premix | Potassium Citrate 2.5 g/Kg | Potassium Citrate 2.5 g/Kg |
|  | Potassium Dihydrogen Phosphate 6.9 g/Kg | Potassium Dihydrogen Phosphate 6.9 g/Kg |
|  | Potassium Sulphate 1.6 g/Kg | Potassium Sulphate 1.6 g/Kg |
|  | Choline Chloride (75%) 2.5 g/Kg | Choline Chloride (75%) 2.5 g/Kg |
|  | AIN93 Vitamins 10 g/Kg | AIN93 Vitamins 10 g/Kg |

**Table S1.** Composition of diets fed to mice in this study. NC – normal chow; HF – high fat; HGI – high glycemic index.
