# Supplementary figures and images for: Gastrointestinal Helminth Infection Improves Insulin Sensitivity, Decreases Systemic Inflammation, and Alters the Composition of Gut Microbiota in Distinct Mouse Models of Type 2 Diabetes
Source: Front Endocrinol (Lausanne). 2021 Feb 5;11:606530. doi: 10.3389/fendo.2020.606530 (PMC7892786; doi:10.3389/fendo.2020.606530)

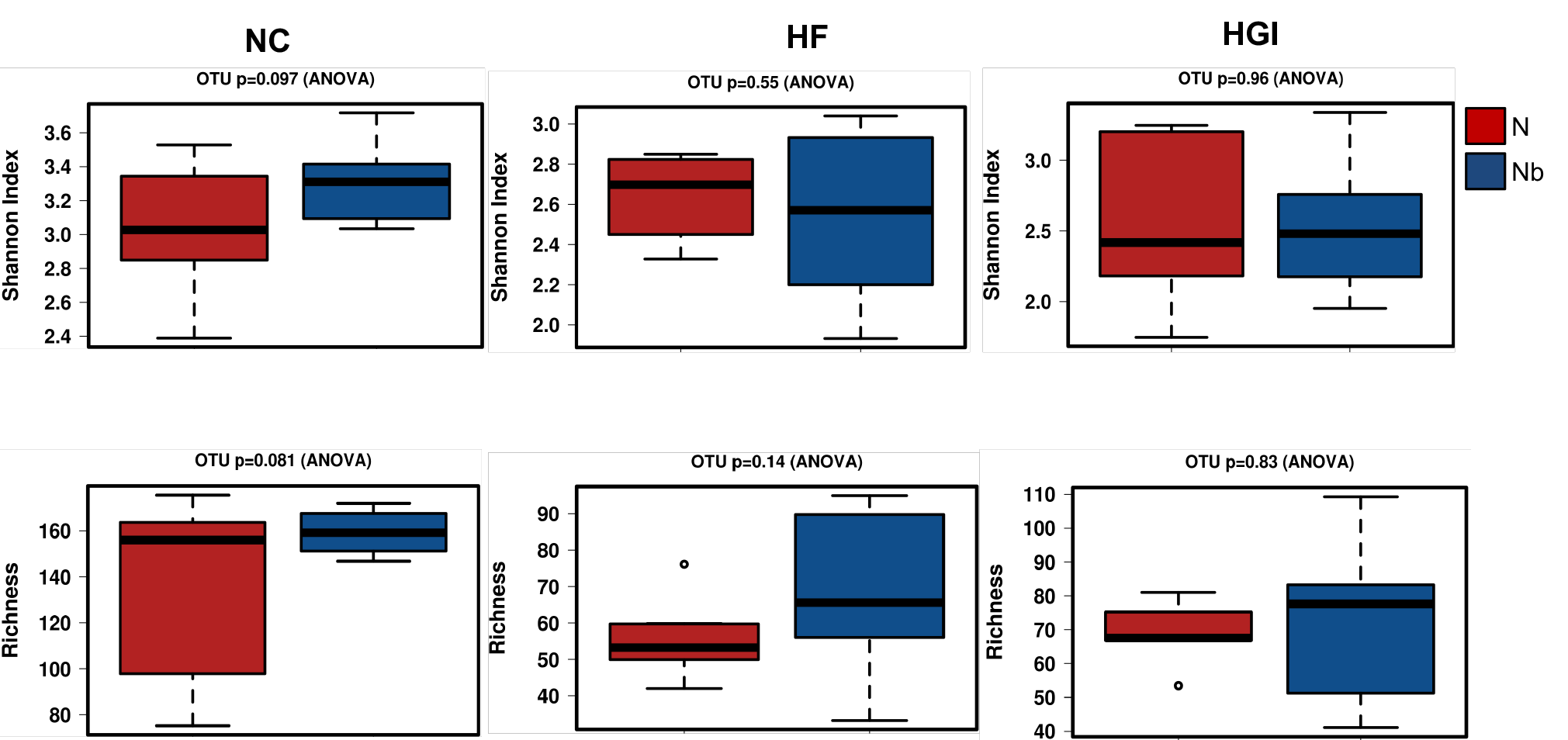

Supplement: Supplementary Figure 1 — Shannon index and richness in the small intestine of Nippostrongylus brasiliensis (Nb) infected and uninfected (naïve, N) C57BL/6 mice fed on normal control (NC), high fat (HF) or high glycaemic index (HGI) diet. Mice were infected once monthly from 6 weeks of age with Nb infective larvae. P values are based on multiple linear regression and are representative of 2 experiments where n = 5/group. [file Image_1.tif]

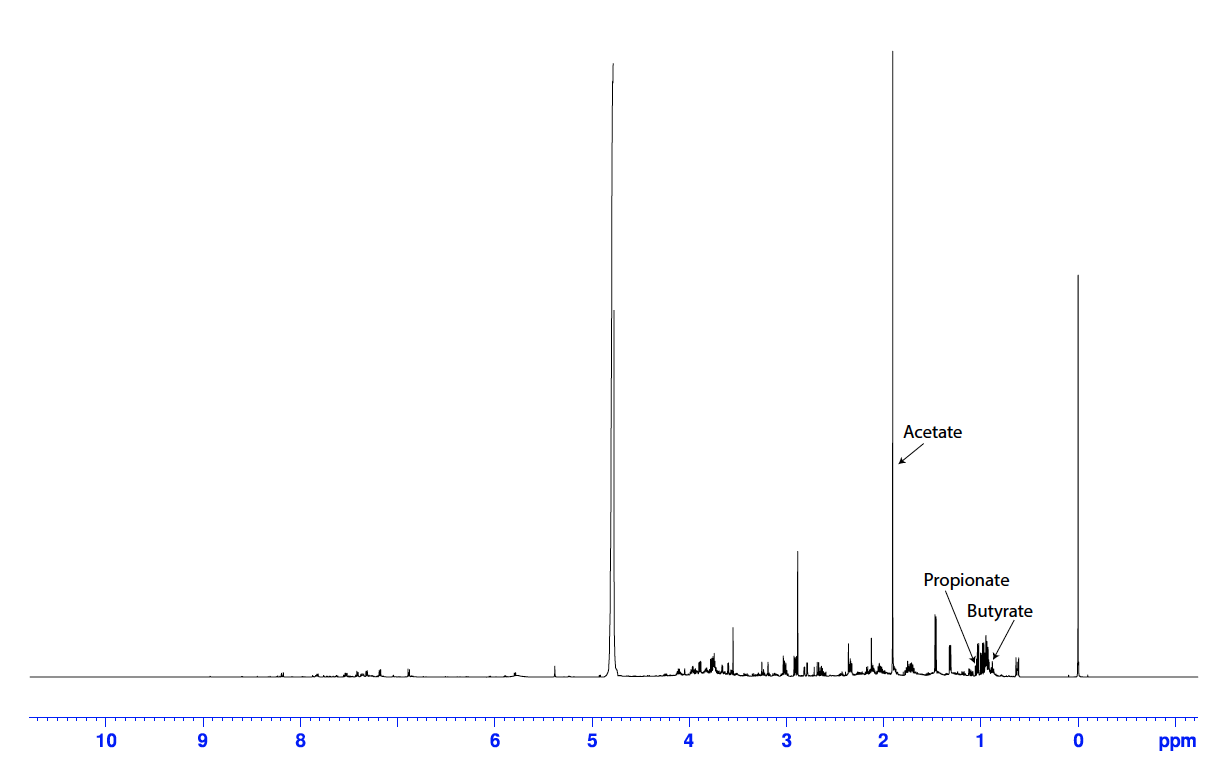

Supplement: Supplementary Figure 2 — Example 1H NMR spectra of acetate, propionate and butyrate faecal extract used in this study. [file Image_2.tif]
